# Hair Dyes Resorcinol and Lawsone Reduce Production of Melanin in Melanoma Cells by Tyrosinase Activity Inhibition and Decreasing Tyrosinase and Microphthalmia-Associated Transcription Factor (MITF) Expression

**DOI:** 10.3390/ijms16011495

**Published:** 2015-01-09

**Authors:** Shu-Mei Lee, Yi-Shyan Chen, Chih-Chien Lin, Kuan-Hung Chen

**Affiliations:** 1 Department of Cosmetic Science and Management, Mackay Medicine, Nursing and Management College, 92 Shengjing Road, Beitou, Taipei 11260, Taiwan; 2 Department of Cosmetic Science, Providence University, 200, Sec. 7, Taiwan Boulevard, Shalu Dist., Taichung 43301, Taiwan; E-Mails: yishyan@gm.pu.edu.tw (Y.-S.C.); chchlin@pu.edu.tw (C.-C.L.); happyalan12@gmail.com (K.-H.C.)

**Keywords:** ammonium persulfate, hair dye, lawsone, melanin, microphthalmia-associated transcription factor (MITF), resorcinol, sodium persulfate, tyrosinase

## Abstract

Hair coloring products are one of the most important cosmetics for modern people; there are three major types of hair dyes, including the temporary, semi-permanent and permanent hair dyes. The selected hair dyes (such as ammonium persulfate, sodium persulfate, resorcinol and lawsone) are the important components for hair coloring products. Therefore, we analyzed the effects of these compounds on melanogenesis in B16-F10 melanoma cells. The results proved that hair dyes resorcinol and lawsone can reduce the production of melanin. The results also confirmed that resorcinol and lawsone inhibit mushroom and cellular tyrosinase activities *in vitro*. Resorcinol and lawsone can also downregulate the protein levels of tyrosinase and microphthalmia-associated transcription factor (MITF) in B16-F10 cells. Thus, we suggest that frequent use of hair dyes may have the risk of reducing natural melanin production in hair follicles. Moreover, resorcinol and lawsone may also be used as hypopigmenting agents to food, agricultural and cosmetic industry in the future.

## 1. Introduction

Hair is an essential feature that plays a key role in self-perception. Sometimes, it also expresses people’s personality. Hair style can be created and modified through hair cosmetics, including hair conditioning, hair straightening, hair waving and hair coloring cosmetic products [1]. Hair graying is a natural part of aging, which occurs in approximately 50% of the people over 50 years old. Therefore, the product of hair coloring is the one of the most important cosmetics for modern people [2].

**Figure 1:**
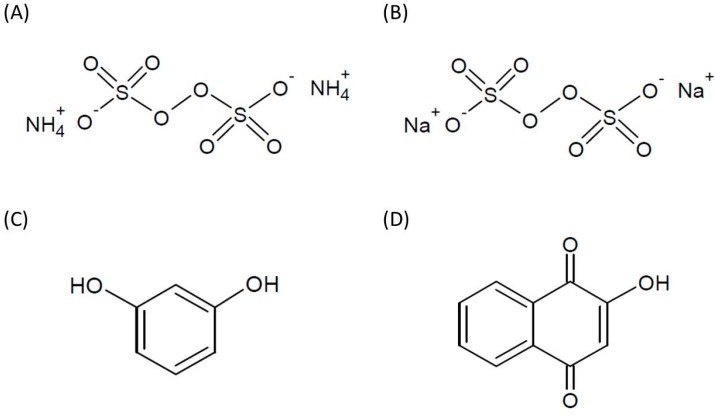
Chemical structures of (**A**) ammonium persulfate; (**B**) sodium persulfate; (**C**) resorcinol and (**D**) lawsone.

For hair coloring cosmetics, there are three major types of hair dyes, namely, the temporary, semi-permanent and permanent hair dyes. In general, temporary hair dyes are water-soluble dyes of high molecular weight, which cannot penetrate the hair shaft. The dyes are only temporarily dropped on hair and can be washed off via normal cleaning procedure [3]. Some of the temporary hair dyes can be obtained from natural resources, such as henna from *Lawsonia inermis* Linn [4]. Semi-permanent hair dyes are generally synthetic and are typically composed of relatively low molecular weight coal tar materials. These dyes can diffuse freely in and out of the cortex and remain on the hair longer than a temporary dye [1,5]. Permanent hair dyes are the most frequently used on hair; this is due to the persistence and darkness effects it has on the hair. For permanent hair dyes, the oxidation procedures are dependent on three main components, *i.e.*, primary intermediates, couplers and oxidants. Thus, during the hair dying process, the hair must firstly be bleached with a mixture containing ammonium/sodium persulfate and hydrogen peroxide [1,6]. After the hairs are bleached, the dye formation reactions will occur by primary intermediates (such as *p*-phenylenediamines) with couplers to attain the final hair color. The commonly used couplers are resorcinol and *m*-aminophenols [7]. These steps may have adverse effects that can damage both the hair and the skin cells. Therefore, the effects of such components of hair dyes on cells are very important for their safety. The chemical structures of selected hair dyes are shown in Figure 1.

Humans and animals generally produce melanin for protection against exposure to ultraviolet (UV) radiation. Melanogenesis (melanin synthesis) is an oxidative chemical reaction, which depends on an important rate-limiting enzyme (tyrosinase) within melanocytes in the parts of skin and hair follicles. Besides, tyrosinase activities are widely distributed in all fields of life, from microorganisms to mammals [8,9]. Tyrosinase is the key enzyme that catalysts tyrosine and dihydroxyphenylalanine (DOPA) into dopaquinone [10]. In addition, microphthalmia-associated transcription factor (MITF) in its phosphorylated active form regulates the expression of tyrosinase-related enzymes. Therefore, tyrosinase and MITF are two of the important regulation points for melanogenesis in cells [11,12].

Melanocytes are responsible for the cutaneous synthesis and distribution of melanin. Melanocytes are localized in the hair between cells covering the hair papilla in the hair bulb; the stem cells for melanocytes are placed in the region of the hair bulge. The ratio of melanocytes to keratinocytes in hair follicles is 1:5; it is more dense than the ratio of 1:10 in the epidermis [13]. For hair graying, several mechanisms acting at different levels and follicular locations ranging from melanocyte stem cells defects to follicular melanocyte death have been proposed. A major issue common to these processes is oxidative damage [14]. Therefore, using hair dyes to restore or to modify hair color may enhance hair-graying state through oxidative damage from hair dyes.

In the present study, we analyzed the effects of selected hair dyes (namely, ammonium persulfate, sodium persulfate, resorcinol and lawsone) on the melanogenesis in B16-F10 melanoma cells in order to confirm the potential of hair dyes on hypo-pigmentation effects. We also analyzed the effect of selected hair dyes on cellular tyrosinase activity, that is, the tyrosinase and MITF protein levels in B16-F10 cells, in order to verify the possible mechanisms of selected hair dye effects on the production of melanin in cells.

## 2. Results and Discussion

### 2.1. Effects of Hair Dyes on Cell Viability of B16-F10 Cells

**Figure 2:**
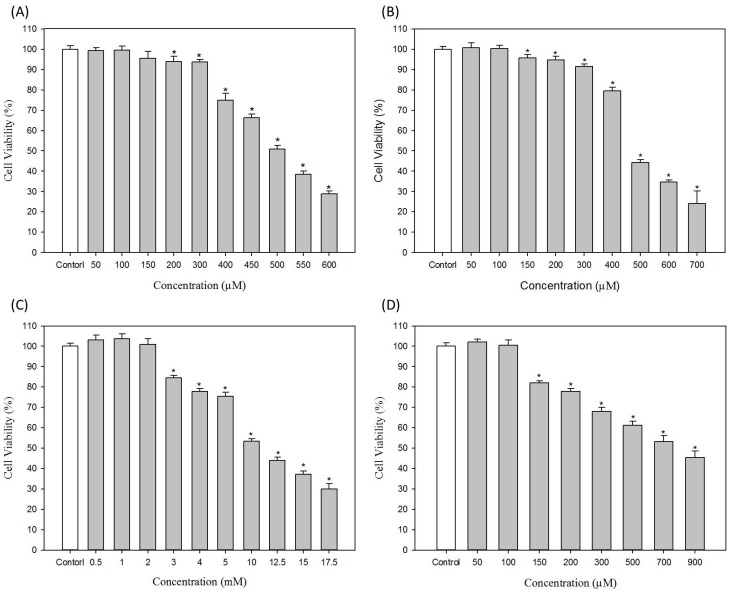
Effects of hair dyes on the cell viability of B16-F10 cells. (**A**) Ammonium persulfate; (**B**) Sodium persulfate; (**C**) Resorcinol and (**D**) Lawsone. Each value is expressed as the mean ± S.E. (*n* = 6), * *p* < 0.05 compared with the control.

In this study, we investigated the effects of commonly used hair dyes on melanogenesis in B16-F10 melanoma cells. The cytotoxicities of ammonium persulfate, sodium persulfate, resorcinol and lawsone on B16-F10 cells were first evaluated; the results are shown in Figure 2. For ammonium persulfate in Figure 2A, the concentrations below 100 μM have no cytotoxicity to B16-F10 cells; 400 μM ammonium persulfate can reduce the cell viability of B16-F10 cells to approximately 75%. The IC_50_ value of ammonium persulfate to B16-F10 cells is 502 ± 3.9 μM. In Figure 2B, the concentrations of sodium persulfate less than 100 μM have no cytotoxicity to B16-F10 cells. The IC_50_ value of sodium persulfate to B16-F10 cells is 524.8 ± 10.2 μM.

For resorcinol, in Figure 2C, the cell viability is not less than 100% if the concentrations of resorcinol are less than 2 mM. The obvious cytotoxic effect was observed when resorcinol concentrations are greater than 3 mM (Figure 2C). The calculated IC_50_ value of resorcinol is 11.1 ± 0.4 mM. For lawsone, the concentrations less than 100 μM have no cytotoxicity to B16-F10 cells (Figure 2D). The IC_50_ value of lawsone to B16-F10 cells is 747.0 ± 6.9 μM. In the experiments of the study, the non-cytotoxic concentrations of ammonium persulfate, sodium persulfate, resorcinol and lawsone used were 100 μM, 100 μM, 2 mM and 100 μM, respectively.

### 2.2. Effects of Hair Dyes on the Melanin Content of B16-F10 Cells

**Figure 3:**
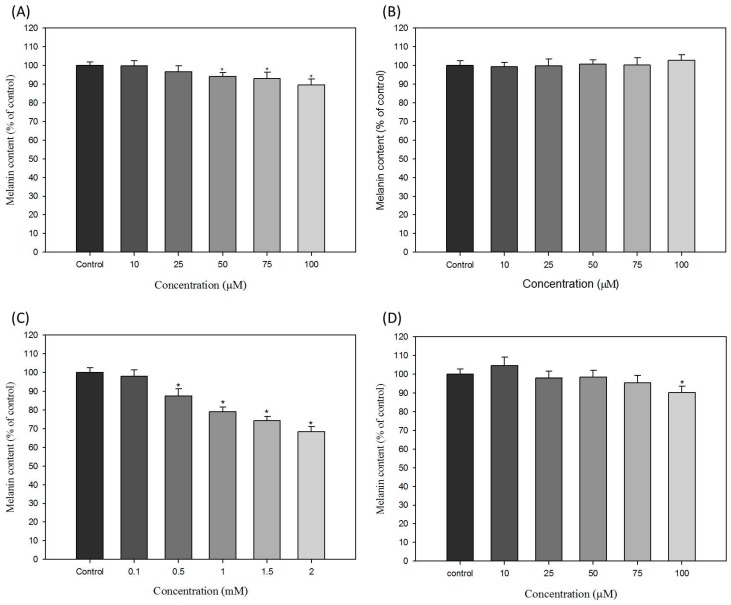
Effects of hair dyes on the melanin content of B16-F10 cells. (**A**) ammonium persulfate; (**B**) sodium persulfate; (**C**) resorcinol and (**D**) lawsone. Each value is expressed as the mean ± S.E. (*n* = 6). * *p* < 0.05 compared with the control.

To evaluate the effect of hair dyes with ammonium persulfate, sodium persulfate, resorcinol and lawsone on the melanogenesis of B16-F10 cells, we analyzed the function of selected hair dyes on the melanin content in B16-F10 cells. The results are shown in Figure 3; the level of melanin content in untreated control B16-F10 cells is defined as 100%. Ammonium persulfate slightly reduces melanin production in a dose-dependent manner in B16-F10 cells. At the concentration of 100 μM of ammonium persulfate, melanin content in B16-F10 cells decreases to approximately 90% of the control (Figure 3A). In Figure 3B, sodium persulfate has no effects on melanin production in all tested concentrations.

In Figure 3C, resorcinol obviously inhibits the production of melanin in a dose-dependent manner in B16-F10 cells. From 0.5 to 2 mM resorcinol treatments, melanin levels decreased from 89.8% to 68.3% of the control. The results showed that resorcinol at higher concentrations have potent effects to reduce melanogenesis in B16-F10 cells. For lawsone, the results are shown in Figure 3D. The melanin content was reduced to approximately 90.3% of the control under treatment of 100 μM lawsone. Although the levels of melanin in other treatment conditions of lawsone are not clearly changed, the reducing effect of lawsone on melanin production is still recognized in this result (Figure 3D).

### 2.3. In Vitro Effects of Hair Dyes on the Activity of Mushroom Tyrosinase and B16-F10 Cellular Tyrosinase

**Figure 4:**
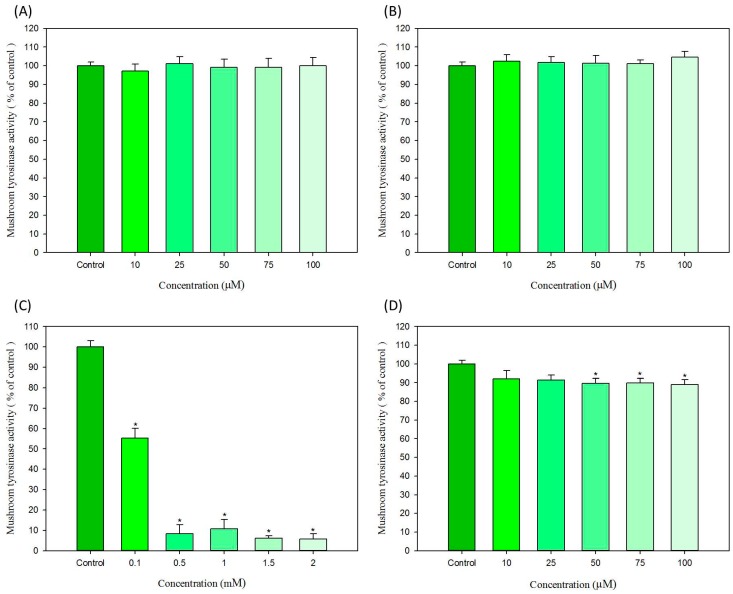
*In vitro* effects of hair dyes on the activity of mushroom tyrosinase. (**A**) ammonium persulfate; (**B**) sodium persulfate; (**C**) resorcinol and (**D**) lawsone. Each value is expressed as the mean ± S.E. (*n* = 6). * *p* < 0.05 compared with the control.

**Figure 5:**
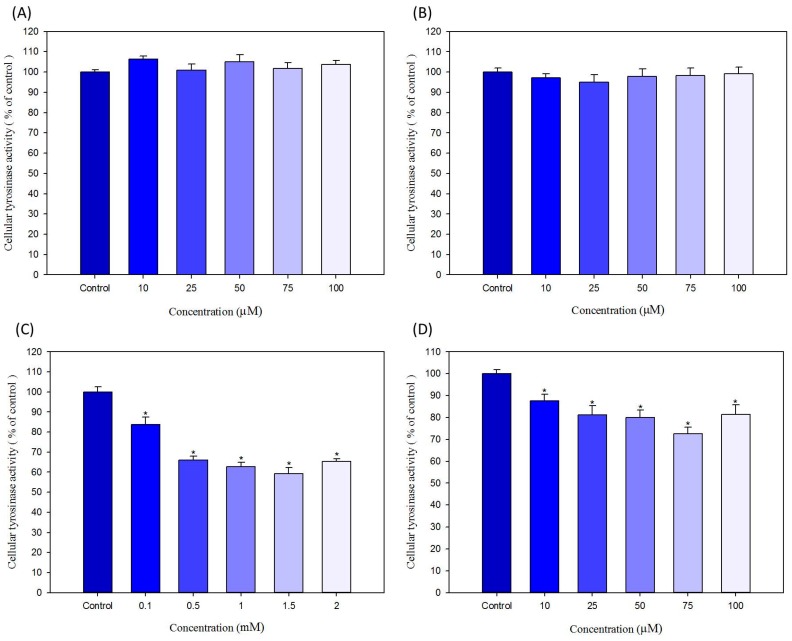
*In vitro* effects of hair dyes on the activity of B16-F10 cellular tyrosinase. (**A**) ammonium persulfate; (**B**) sodium persulfate; (**C**) resorcinol and (**D**) lawsone. Each value is expressed as the mean ± S.E. (*n* = 6). * *p* < 0.05 compared with the control.

To test whether the reducing effect of selected hair dyes on melanogenesis is mediated by direct inhibition of tyrosinase activity, the hair dyes were used to check the *in vitro* anti-tyrosinase activity on both purified mushroom tyrosinase and extracted B16-F10 cellular tyrosinase; the results are shown in Figure 4 and Figure 5, respectively.

For mushroom tyrosinase, ammonium persulfate and sodium persulfate have no effects on the tyrosinase activity. As shown in Figure 4A,B, all treatment concentrations, both ammonium persulfate and sodium persulfate cannot influence the activity of mushroom tyrosinase *in vitro*. In Figure 4C, resorcinol demonstrated the potent inhibition effect on mushroom tyrosinase activity in a dose-dependent manner. From concentrations of 0.5 to 2 mM, resorcinol represses the mushroom tyrosinase activity to only about 10% of the control. As show in Figure 4D, lawsone can only inhibit the activity of mushroom tyrosinase approximately 10% of control at the concentrations of 50 μM. Although the *in vitro* effect of lawsone on mushroom tyrosinase activity is not quite strong, we can still suppose that lawsone has a slight inhibition effect for mushroom tyrosinase (Figure 4D).

For B16-F10 cellular tyrosinase, ammonium persulfate and sodium persulfate also did not inhibit the activity of the tyrosinase. In Figure 5A,B, at all treated conditions, both ammonium persulfate and sodium persulfate did not affect the activity of B16-F10 cellular tyrosinase *in vitro*. These results are almost in correlation with those depicted in Figure 4A,B. In contrast, resorcinol and lawsone evidently repress the activity of B16-F10 cellular tyrosinase. Resorcinol (1.5 mM) and lawsone (75 μM) decreased cellular tyrosinase activities to 60.2% and 71.9% of control, respectively (Figure 5C,D). Although the highest concentrations of resorcinol and lawsone did not have the most potent anti-tyrosinase activity on B16-F10 cellular tyrosinase, the effects of resorcinol and lawsone are still clearly demonstrated as shown in Figure 5C,D.

The chemical structure of resorcinol is similar to the hypopigmentation compound, hydroquinone. Therefore, it is expected that resorcinol possess the ability to inhibit tyrosinase activity [15]. Some previous studies have investigated the *in vitro* inactivation effects of resorcinol and other resorcinol derivatives on mushroom tyrosinase (*Agaricus bisporus*). The results revealed that the resorcinol substrate is oxidized via the mono-oxygenase route of tyrosinase leading to a hydroxy intermediate that undergoes deprotonation, which results in irreversible elimination of Cu(0) from the active site [16,17]. However, no previous study has tested the effects of lawsone on melanogenesis and on tyrosinase activity. Therefore, this study is the first to demonstrate the inhibition activity of lawsone on melanin production and tyrosinase activity. The results showed that lawsone suppress both the activities of mushroom tyrosinase and B16-F10 cellular tyrosinase *in vitro* (Figure 3D, Figure 4D and Figure 5D).

### 2.4. Effects of Hair Dyes on the Cellular Tyrosinase Activity in B16-F10 Cells

**Figure 6:**
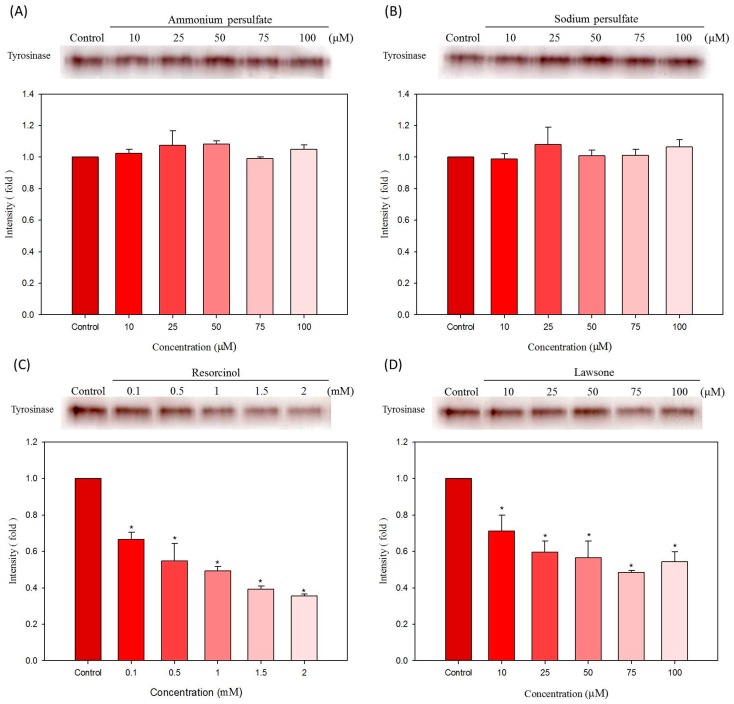
Effects of hair dyes on the cellular tyrosinase activity in B16-F10 cells. (**A**) ammonium persulfate; (**B**) sodium persulfate; (**C**) resorcinol and (**D**) lawsone. Each value is expressed as the mean ± S.E. (*n* = 3). * *p* < 0.05 compared with the control.

To further examine the anti-melanogenesis functions of the selected hair dyes, we analyzed the effects of the hair dyes on tyrosinase in B16-F10 Cells using a zymographic assay. The B16-F10 cells were firstly treated with various concentrations of the samples for 24 h, and then the cellular proteins were extracted to analyze the tyrosinase activity of protein samples. This analysis explains the effect of tested compound on the cellular tyrosinase within cells. The results are shown in Figure 6. For ammonium persulfate and sodium persulfate, the compounds did not significantly affect the formation of melanin-containing dark bands in all tested samples from B16-F10 cells (Figure 6A,B). The results revealed that ammonium persulfate and sodium persulfate do not reduce the activity of cellular tyrosinase. In addition, resorcinol was found to decrease the dark bands in a dose-dependent manner; the inhibition ratio is approximately 0.6-fold of control at the condition of 2 mM resorcinol treatment (Figure 6C). Similar to the results of resorcinol, lawsone was found to attenuate the melanin-containing dark bands in all treatments. At the condition of 70 μM lawsone treatment, the band intensity decreased to approximately 0.45-fold of control (Figure 6D). Therefore, lawsone has the ability to influence tyrosinase activity in B16-F10 cells.

Ammonium persulfate was initially used to bleach the hair in the hair dyeing procedure. Therefore, ammonium persulfate may have ability to attenuate the darkness of the formed melanin [18]. We found that ammonium persulfate can reduce the production of melanin in B16-F10 cells (Figure 3A), however, the activities of cellular tyrosinase may not directly or indirectly be affected by ammonium persulfate (Figure 5A and Figure 6A). Thus, we examined and verified the bleaching activity of ammonium persulfate on extracted melanin *in vitro*. The result demonstrated that ammonium persulfate can directly decrease the darkness of extracted melanin (data not show). This explains why ammonium persulfate reduces melanin content in treated cells, and had no influence on the activity of cellular tyrosinase.

### 2.5. Effects of Hair Dyes on the Protein Levels of Tyrosinase and MITF in B16-F10 Cells

**Figure 7:**
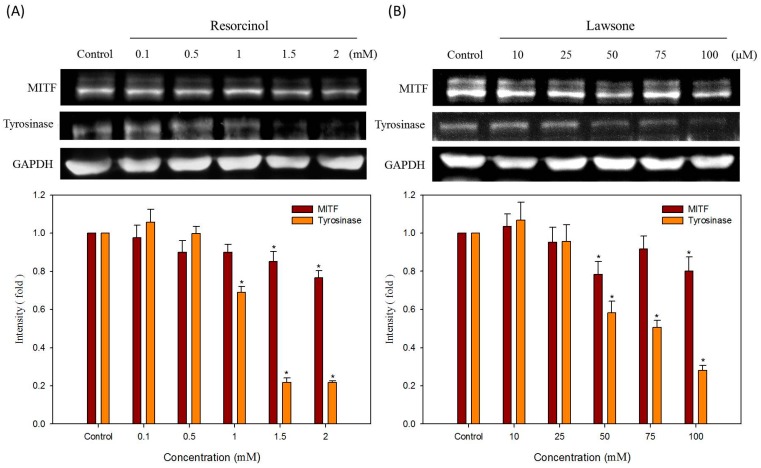
Effects of hair dyes (**A**) resorcinol and (**B**) lawsone on the protein levels of MITF and tyrosinase in B16-F10 cells. The protein level of GAPDH is used as an internal control. Each value is expressed as the mean ± S.E. (*n* = 3). * *p* < 0.05 compared with the control.

To further check the mechanism of resorcinol and lawsone on the inhibition of melanin production, we tested the levels of the most important melanogenesis regulation proteins, MITF and tyrosinase, in resorcinol and lawsone treated B16-F10 cells. Results are shown in Figure 7; the quantitative results are shown in lower figures. The glyceraldehyde-3-phosphate dehydrogenase (GAPDH) protein was used as an internal control for normalization. For resorcinol, GAPDH protein levels are equal in all samples, but MITF are slightly decreased with the increasing concentrations of resorcinol. At 2 mM resorcinol treatment condition, the level of MITF diminished to the lowest amount (Figure 7A). Tyrosinase protein levels are significantly reduced with the treatments of resorcinol. Tyrosinase levels are clearly downregulated by resorcinol if the concentrations are higher than 1.5 mM (Figure 7A). For lawsone, the result is shown in Figure 7B; GAPDH proteins are also equivalent in all treatments. However, both MITF and tyrosinase proteins in B16-F10 cells are evidently repressed by the treatments of lawsone. The reducing effects of lawsone are noticeable when its concentrations are higher than 50 μM (Figure 7B). Therefore, we suggest that resorcinol and lawsone have functions to reduce the protein levels of MITF and tyrosinase in B16-F10 cells.

Many compounds inhibit the production of melanin through their direct anti-tyrosinase activity, which include arbutin, deoxyArbutin, kojic acid and quercetin [19,20]. In contrast, several other compounds downregulate the expression of melanogenesis-related proteins to repress the melanin synthesis in melanocytes, such as lipoic acid, retinoic acid, fenofibrate and hispolon [21,22,23,24]. However, some hypopigmentation agents (such as resveratrol) can inhibit both the activity of cellular tyrosinase and the protein expressions of tyrosinase and MITF [25]. The inhibition of tyrosinase activity by resveratrol was explained by both direct tyrosinase inhibition, through acting as a substrate for tyrosinase and a post-transcriptional effect that reduced the amount of the fully processed tyrosinase [26]. Thus the dual functions of such compounds may provide a potent anti-melanogenic effect. Accordingly, our results showed that resorcinol and lawsone inhibit both cellular tyrosinase activity as well as MITF and tyrosinase expressions in B16-F10 cells.

## 3. Experimental Section

### 3.1. Materials

Ammonium persulfate, sodium persulfate, resorcinol, lawsone, mushroom tyrosinase, l-DOPA, dimethyl sulfoxide (DMSO), sodium hydroxide (NaOH), sodium dodecyl sulfate (SDS) and other chemicals were purchased from Sigma-Aldrich (St. Louis, MO, USA). Dulbecco’s modified Eagle’s medium (DMEM), fetal bovine serum (FBS), penicillin, streptomycin and trypsin-EDTA were purchased from Gibco BRL/Invitrogen (Carlsbad, CA, USA). The 3-(4,5-dimethylthiazol-2-yl)-2,5-diphenyl tetrazolium bromide (MTT) was purchased from Affymetrix/USB (Cleveland, OH, USA). The anti-MITF, anti-tyrosinase and anti-glyceraldehyde-3-phosphate dehydrogenase (GAPDH) antibodies were purchased from Santa Cruz Biotechnology (Santa Cruz, CA, USA). Protease inhibitor cocktail was purchased from Abcam Inc. (Cambridge, MA, USA). Deionized distilled water (ddH_2_O) for solutions and buffers was obtained from the Milli-Q system (Millipore, Bedford, MA, USA).

### 3.2. Cell Line and Cell Culture

The B16-F10 mouse melanoma cells (BCRC 60031) were purchased from the Food Industry Research and Development Institute (FIRDI, Hsinchu, Taiwan). The B16-F10 cells were cultured in DMEM and 10% FBS, 2 mM glutamine, 100 mg/mL streptomycin and 100 U/mL penicillin were added. The cells were sustained in a humidified incubator with 5% CO_2_ at 37 °C. Every three to four days, the cells were sub-cultured to maintain logarithmic growth [27].

### 3.3. MTT Assay for Cell Viability

B16-F10 cells were seeded in 96-well plates at a cell density of 5 × 10^3^ cells/well by DMEM medium supplemented with 10% FBS for 24 h. After cell seeding, the prepared cells were subsequently treated with different concentrations of samples for 24 h. Then, 100 μL (0.5 mg/mL) of MTT solution was added to cells and incubated at 37 °C for 30 min, then washed twice with phosphate-buffered saline (PBS). Lastly, the cells were lysed with 100 μL of DMSO, and the absorbance was measured spectrophotometrically at 540 nm using an ELISA reader. The inhibitory concentration value at 50% cell viability is defined as IC_50_ [28].

### 3.4. Melanin Content Analysis

For melanin content analysis, B16-F10 cells with cell density of 8 × 10^4^ cells/well were incubated in 6-well plates for 24 h. The cells were then treated with various concentrations of the test samples for 24 h. Thereafter, the cells were dissolved in 120 μL of 1 N NaOH for 1 h at 65 °C to solubilize the melanin. The total amount of melanin in each cell suspension was determined by recording the absorbance at 405 nm. The melanin content was calculated and corrected for cell number [29].

### 3.5. In Vitro Tyrosinase Activity Assays

For the *in vitro* tyrosinase activity assays, each 60-μL test sample was mixed with 10 μL of l-DOPA (10 mM). Then, 40 μL of mushroom tyrosinase solution (100 units/mL) or B16-F10 cellular tyrosinase (800 μg of cellular protein) were added to the mixture, which was then incubated for 25 min at 37 °C. The spectrophotometric analysis was performed at 475 nm and the formation of DOPAchrome was calculated as the percentage of enzyme activity [22].

### 3.6. Zymographic Analysis for Cellular Tyrosinase

The B16-F10 cells with cell density of 1 × 10^6^ cells/well were incubated in 10 cm dish for 24 h. The cells were then treated with various concentrations of the samples for 24 h. After treatments, the cells were sonicated with 200 μL lysis buffer containing a protease inhibitor cocktail at 4 °C for 20 min, and the lysates were collected and then quantified. Proteins samples of 100 μg were mixed in sample buffer without β-mercaptoethanol and separated using 10% SDS PAGE. The gel was rinsed twice in 200 mL of 100 mM sodium phosphate buffer (pH 6.8) and equilibrated with gentle shaking at room temperature. Thereafter, the gel was transferred into 200 mL of a staining solution containing the rinse buffer supplemented with 10 mM l-DOPA and then incubated in the dark at 37 °C for 10 min. The cellular tyrosinase activity was imagined in the gel as a melanin-containing dark band. The bands were scanned and quantified by determining the optical densities by the VIpro Platinum 1.1 software package (Version 12.9; UVItec, Cambridge, UK) [30].

### 3.7. Western Blot Analysis

The B16-F10 cells with cell density of 3 × 10^5^ cells/well were seeded in 6-well plates for 24 h. The cells were then treated with various concentrations of the test samples for 24 h. After treatments, the cells were sonicated with a 200-μL lysis buffer containing a protease inhibitor cocktail at 4 °C for 20 min and the lysates were collected and then quantified. For western blotting, each well was loaded with 20 ng of protein and resolved by 10% SDS PAGE and then electro-transferred on to a polyvinylidene difluoride (PVDF) membrane using the Bio-Rad MiniProtean II apparatus (Bio-Rad Laboratories, Carlsbad, CA, USA). The blots were then incubated with an anti-MITF, anti-tyrosinase or anti-GAPDH antibody as the primary antibody, and the immune complexes were visualized using an ECL reagent (Millipore, Billerica, MA, USA) [31].

### 3.8. Statistical Analysis

The quantitative data were analyzed by Student’s *t*-tests and presented as the mean ± standard error (S.E.) of at least three independent experiments. Calculated *p*-values less than 0.05 were considered to be significant.

## 4. Conclusions

In summary, we analyzed the effects of chosen hair dyes, ammonium persulfate, sodium persulfate, resorcinol and lawsone, on the melanogenesis in B16-F10 cells. The results demonstrated that the hair dyes resorcinol and lawsone have the ability to reduce the production of melanin. Also, we confirmed that resorcinol and lawsone could both inhibit mushroom and cellular tyrosinase activities. Resorcinol and lawsone can downregulate the expression of tyrosinase and MITF proteins in B16-F10 cells. Therefore, in this study, our results indicate that some commonly used hair dyes may have the risk to decrease the naturally produced melanin in melanocyte of hair follicles. Moreover, the dual functions of such compounds may provide a potent anti-melanogenic effect. Thus, we suggest that resorcinol and lawsone may have the potential to be effective hyperpigmentation agents in the food, agricultural and cosmetic industries.
